# Chromosome-scale genome assembly reveals insights into the evolution and ecology of the harmful algal bloom species *Phaeocystis globosa* Scherffel

**DOI:** 10.1016/j.isci.2024.110575

**Published:** 2024-07-25

**Authors:** Nansheng Chen, Qing Xu, Jianan Zhu, Huiyin Song, Liyan He, Shuya Liu, Xiuxian Song, Yongquan Yuan, Yang Chen, Xihua Cao, Zhiming Yu

**Affiliations:** 1 CAS Key Laboratory of Marine Ecology and Environmental Sciences, Institute of Oceanology, Chinese Academy of Sciences, Qingdao 266071, China; 2 Laboratory of Marine Ecology and Environmental Science, Qingdao National Laboratory for Marine Science and Technology, Qingdao 266200, China; 3 Center for Ocean Mega-Science, Chinese Academy of Sciences, Qingdao 266071, China; 4 Department of Molecular Biology and Biochemistry, Simon Fraser University, 8888 University Drive, Burnaby, BC V5A 1S6, Canada; 5 College of Life Science and Technology, Huazhong Agricultural University, Wuhan 430070, China; 6 College of Marine Science, University of Chinese Academy of Sciences, Beijing 10039, China

**Keywords:** Environmental science, Ecology, Microbiology, Microbial genomics, Evolutionary ecology, Aquatic biology, Genomics

## Abstract

The phytoplankton *Phaeocystis globosa* plays an important role in sulfur cycling and climate control, and can develop harmful algal blooms (HABs). Here we report a chromosome-scale reference genome assembly of *P. globosa*, which enable in-depth analysis of molecular underpinnings of important ecological characteristics. Comparative genomic analyses detected two-rounds of genome duplications that may have fueled evolutionary innovations. The genome duplication may have resulted in the formation of dual HiDP and LoDP dimethylsulphoniopropionate (DMSP) biosynthesis pathways in *P. globosa*. Selective gene family expansions may have strengthened biological pathways critical for colonial formation that is often associated with the development of algal blooms. The copy numbers of rhodopsin genes are variable in different strains, suggesting that rhodopsin genes may play a role in strain-specific adaptation to ecological factors. The successful reconstruction of the *P. globosa* genome sets up an excellent platform that facilitates in-depth research on bloom development and DMSP metabolism.

## Highlights

• The first chromosome-level assembly of Phaeocystis globosa genome was constructed
• Two rounds of genome duplications were detected in the evolution of P. globosa
• Annotation of nine copies of full-length ribosomal operons in two chromosomes
• Variations of rhodopsin genes may help ecological adaptation

## Graphical abstract

**Figure:**
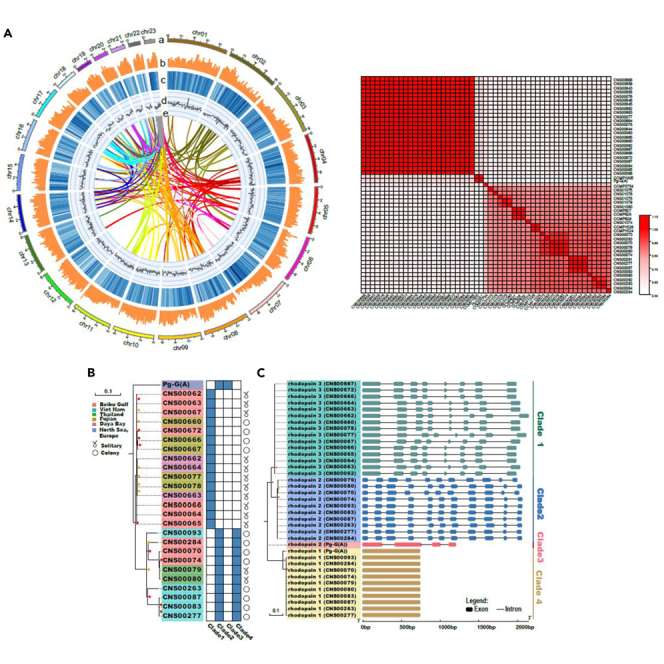

## Introduction

*Phaeocystis* Lagerheim is a cosmopolitan haptophyte genus that thrives in ocean regions ranging from poles to tropics and from coastal to open ocean waters.^1^ *Phaeocystis* is responsible for ∼10% of the annual global primary productivity,^1^ a major contributor to carbon cycling,^2^^,^^3^ and important for sulfur cycling and climate control.^4^^,^^5^ *Phaeocystis globosa* in particular has been demonstrated to be critical in dimethylsulphoniopropionate (DMSP) biosynthesis and in producing the volatile catabolite dimethylsulphide (DMS), which can be oxidized into cloud condensation nuclei in the atmosphere to affect climate.^4^^,^^5^^,^^6^^,^^7^ Recent characterization of two major groups of DMSP producers, high DMSP producers and low DMSP producers,^8^ which have been proposed to correspond to two distinct ecological roles for DMSP.^9^ Nevertheless, molecular mechanisms of DMSP metabolism in *P. globosa* remain currently uncharacterized. Under certain environmental conditions, *Phaeocystis* genus, which encompasses six species including three species capable of developing blooms: *P. globosa*, *P. antarctica*, and *P. pouchetti*,^9^^,^^10^^,^^11^^,^^12^ have been found to develop harmful algal blooms (HABs) in many ocean regions including the North Sea^1^^,^^13^^,^^14^ and China. In China, since the *P. globosa* HABs were first recorded in 1997 in southeast China,^15^ they have been found to expand into essentially all coastal regions in China.^11^^,^^12^ Nevertheless, mechanisms driving the development of *P. globosa* blooms still remain poorly understood.

One outstanding feature of *P*. *globosa* is its heteromorphic life history with complex life stages that alternates between mucilaginous colonies of non-motile coccoid cells and different types of free-living solitary cells.^16^^,^^17^^,^^18^^,^^19^^,^^20^^,^^21^^,^^22^ *P*. *globosa* cells are 3–10 μm in size during the free-living solitary stage, with two flagella and one flagellum-like appendage (haptonema). Colonies often reach several millimeters to centimeters in size, making them visible with the naked eyes, consisting of cells usually without flagella and scales.^23^^,^^24^^,^^25^ *P*. *globosa* colony formation may involve reproduction,^22^ depend symbiotic relationships with bacteria,^26^^,^^27^ inhibit ingestion by zooplankton, protecting the continued growth of cells in the colony,^28^ and play a defensive role for producing colonies.^24^^,^^29^ What molecular mechanisms underpin the formation of *P. globosa* colonies? Transcription studies suggested differential expression of genes important in reallocation of resources associated with forming and maintaining colonies^29^ and glycosaminoglycan (GAGs) accumulation in colony formation.^30^ Nevertheless, without a high-quality reference genome, in-depth and accurate interpretation of transcriptomics results remains inaccurate and challenging.

Genome sizes of *P. globosa* strains collected from different regions showed a remarkable range (109–200 Mb) and these strains could be separated into five groups,^31^ suggesting that *P. globosa* may also have high genetic diversity.^12^^,^^32^^,^^33^^,^^34^ Indeed, we have also uncovered substantial *P. globosa* genetic diversity by applying newly developed molecular markers *pgcp1* and *cox1* with high resolution and high specificity.^35^^,^^36^^,^^37^ Direct assessment of the nuclear genomes of *P. globosa* strains, which is currently lacking, would reveal rich information with the assistance of a reference genome.

In this study, we successfully assembled a high-quality genome sequence of *P. globosa*, which represents the first genome with chromosome-scale continuity for *P. globosa*, using a strain that was isolated in the Beibu Gulf, China and cutting-edge DNA sequencing and genome assembly technologies. Taking advantage of this genome assembly, we have revealed high genetic diversity among *P. globosa* strains, annotated genes important for DMSP biosynthesis and degradation, and gene family expansions that impacted colony formation. The successful reconstruction of the first high-quality *P. globosa* genome sets up a platform for research on bloom development and DMSP metabolism.

## Results

### Construction of the first chromosome-scale genome assembly for *P. globosa*

**Figure 1:**
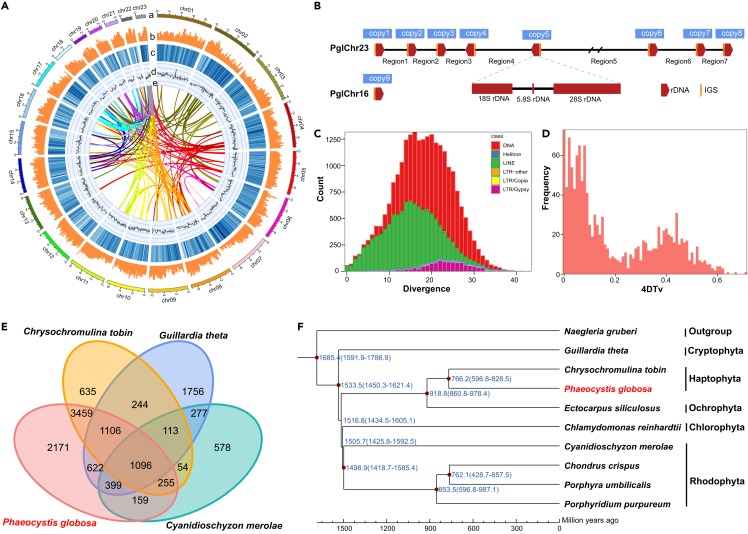
Genomic features of *P. globosa* and comparative analysis of *P. globosa* and other algal species (A) Genomic landscape of the 23 chromosomes of the *P. globosa* genome assembled in this project. Track a represents 23 *P globosa* chromosomes (Mb); Tracks b–d represent distribution of gene density, repeat element density, and GC content, respectively (bin size = 100k); Track e represents syntenic blocks in the *P. globosa* genome. (B) Distribution of nine copies of ribosomal operons in chromosomes *PglChr23* (eight copies) and *PglChr16* (one single copy). Each copy consisted of an 18S rDNA, an internal transcribed spacer (ITS, including 5.8S rDNA), a 28S rDNA, and an IGS. Two adjacent copies are separated by a genomic sequence of various lengths. (C) Divergence distribution of transposable elements. (D) Whole genome duplication (WGD) events are estimated from the 4-fold degenerate synonymous sites of the third codons (4DTv) distance of homologous pairs in syntenic regions of *P. globosa*. (E) Venn diagram for orthologous protein-coding gene clusters in *P. globosa*, *C. tobin*, *G. theta*, and *C. merolae*. (F) Evolutionary analysis of single copy genes in *P. globosa* and selected species.

The *P. globosa* strain CNS00066, which was isolated from water samples collected during a bloom in the Beibu Gulf, China in January, 2019, was sequenced using Illumina and PacBio HiFi platforms, yielding 11.28 Gb and 6.44 Gb data, respectively. The strain CNS00066 was selected among dozens of candidate *P. globosa* strains as the reference strain because of its minimum amount of bacterial contamination (3.85%) (Figure S1). The draft *P. globosa* genome was successfully assembled (Figure 1A) with contig N50 being 0.57 Mb, which was further assembled through Hi-C analysis,^38^ yielding 23 chromosomes (Figure S2) with a scaffold N50 being 6.6 Mb (Table 1). The GC content of *P. globosa* genome was 64.6%, which was similar to that of the haptophte species *Emiliania huxleyi* (65.7%),^39^ but lower than that of the haptophte species *Diacronema lutheri* (73.3%).^12^^,^^40^ Telomeric repeat motif CCCTAA was identified to be enriched at the chromosome ends, suggesting the potential completeness of assembled *P. globosa* chromosomes. Transposable elements (TEs) showed divergence rate of about 20%, with younger TEs being long interspersed nuclear elements (LINEs) and most active TEs being long terminal repeat (LTR)-Gypsys (Figure 1C; Table S2). We successfully uncovered full-length sequences of nine copies ribosomal gene clusters (operons) in the *P. globosa* genome, each of which contains an 18S rDNA, a 5.8S rDNA, a 28S rDNA, and an intergenic spacer (IGS). These operons were found to be loosely clustered on the chromosome *PglChr23* (eight copies) with a single copy found on the chromosome *PglChr16* (Figure 1B). Interestingly, the eight copies of ribosomal operons were not strictly in tandem, but were instead separated by variable genomic sequences (14.4–290.9kb) between adjacent copies.

**Table 1:** Comparison of published haptophyte genomes with *P. globosa*

	*Phaeocystis. globosa*	*Emiliania huxleyi*	*Chrysochromulina tobin*	*Chrysochromulina parva*	*Tisochrysis lutea*	*Diacronema lutheri*
	This study	Read et al. (2013)^39^	Hovde et al. (2015)^41^	Hovde et al. (2019)^42^	Carrier et al. (2018)	Hulatt et al. (2021)^40^
**Assembly features**						

Assembly length (Mb)	129.7	141.7	59.0	65.8	57.7	43.5
Chromosomes number	23	–	–	–	–	–
Scaffolds number	85	7,795	–	–	–	–
Scaffolds N50 (kp)	6601.8	404.8	–	–	–	–
Contigs number	396	16,921	3,412	8,362	–	103
Contig N50 (kp)	569.7	29.7	24.1	16.1	10.6	852.3
GC ratio (%)	64.6	65.7	63.4	63.6	58.7	73.3

**Genome annotation**						

Number of protein-coding genes	32,618	30,569	16,777	28,138	20,582	14,446
Average gene length (bp)	2,888	1,718	1,899	–	–	–
Median gene length (bp)	2,046	1,243	1,404			
Average CDS length (bp)	273	–	–	–	–	–
BUSCO completeness of annotation (%)	80.9	51.8	62.0	72.9	68.3	80.8

### Two rounds of genome duplication may have driven the evolution of *P. globosa*

The *P. globosa* genome was annotated to have 32,618 protein-coding genes (PCGs), including 80.9% benchmarking universal single-copy orthologs (BUSCO) complete genes, which was the highest BUSCO value among all haptophyte species (Table 1; Figure S3). The number of PCGs in *P. globosa* was also the highest among all sequenced haptophyte species, suggesting that *P. globosa* genome might have experienced certain types of genome duplication. To test this hypothesis, we carried out all-against-all PCG comparison, which identified 171 pairs of genome segments of various lengths ranging from 7 kb to 57 kb (average being 14.7 kb and a median being 120 kb) (Figure 1A), containing 1449 homologous gene pairs with high similarity. Further analysis suggested that *P. globosa* genome might have experienced two rounds of segmental genome duplication (Figure 1D), which was supported by calculation of 4DTv and the number of synonymous substitutions per synonymous site (Ks) of homologous gene pairs (Figures 1D and S4). Phylogenetic analysis of 145 single-copy genes shared by 10 algal species showed a close evolutionary relationship between two haptophytes *P. globosa* and *Chrysochromulina tobin* as expected (Figure 1F). These two haptophyte species split at about 766 million years ago (Mya). Comparative analysis of PCGs of four phytoplankton species (including two haptophyte species *P. globosa* and *C. tobin*, one cryptophyte species *Guillardia theta*, and one red algal species *C. merolae*) identified only 1909 gene clusters shared by all species (Figure 1E), suggesting large evolutionary distances among these species. In contrast, two haptophyte species *P. globosa* and *C. tobin* shared 5916 gene families, consistent with their closer evolutionary relationship (Figure 1E).

### DMSP biosynthesis genes and their variations in *P. globosa*

**Figure 2:**
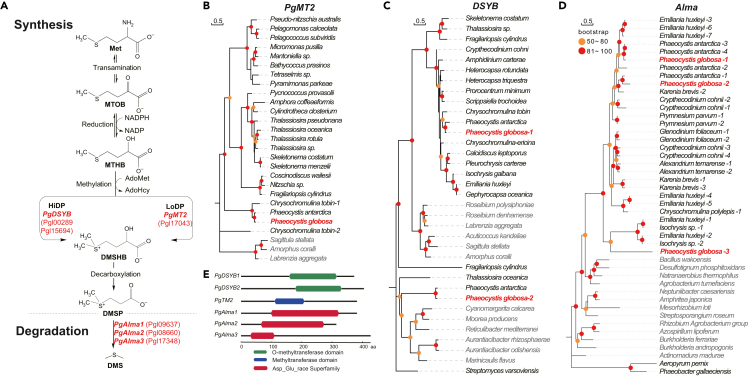
Genes involved in DMSP biosynthesis and degradation in *P. globosa* (A) Key genes in DMSP biosynthesis and degradation in *P. globosa*; (B) Phylogenetic analysis of *PgMT2* genes; (C) Phylogenetic analysis of *DSYB* genes; (D) Phylogenetic analysis Alma genes; (E) Important functional domains identified in *PgDSYB*, *PgTM2*, and *PgAlma*.

Genome annotation identified that *P. globosa* genome encodes dual DMSP biosynthesis pathways HiDP and LoDP, catalyzed by *PgDSYB* (Figure 2A) and *PgMTs* (Figure 2B), respectively. This is unexpected because only a few organisms have been identified to contain both DMSP biosynthesis pathways.^9^ Furthermore, two DSYB genes of the HiDP biosynthesis pathway were found in *P. globosa*, suggesting that the HiDP biosynthesis pathway may be further enhanced in *P. globosa*. In addition to the utilization of a DSYB gene that was shared by many bacteria and algae, *P. globosa* genome encoded an additional copy of the DSYB gene that was also found in *P. antarctica* (Figure 2A), which may have gained via horizontal gene transfer (HGT) from bacteria because most of the homologs were bacterial genes (Figure 2C). Degradation of DMSP was catalyzed by *Alma* genes, whole copy numbers were found to be variable in *P. globosa* strains. While three copies of *Alma* genes (*Alma1*, *Alma2*, and *Alma3*) were identified in many *P. globosa* strains including the reference genome (CNS00066 strain) (Figures 2A and 2D), two copies of *Alma* genes were found in many other *P. globosa* strains.

### Gene family expansion characterized colony formation of *P. globosa*

**Figure 3:**
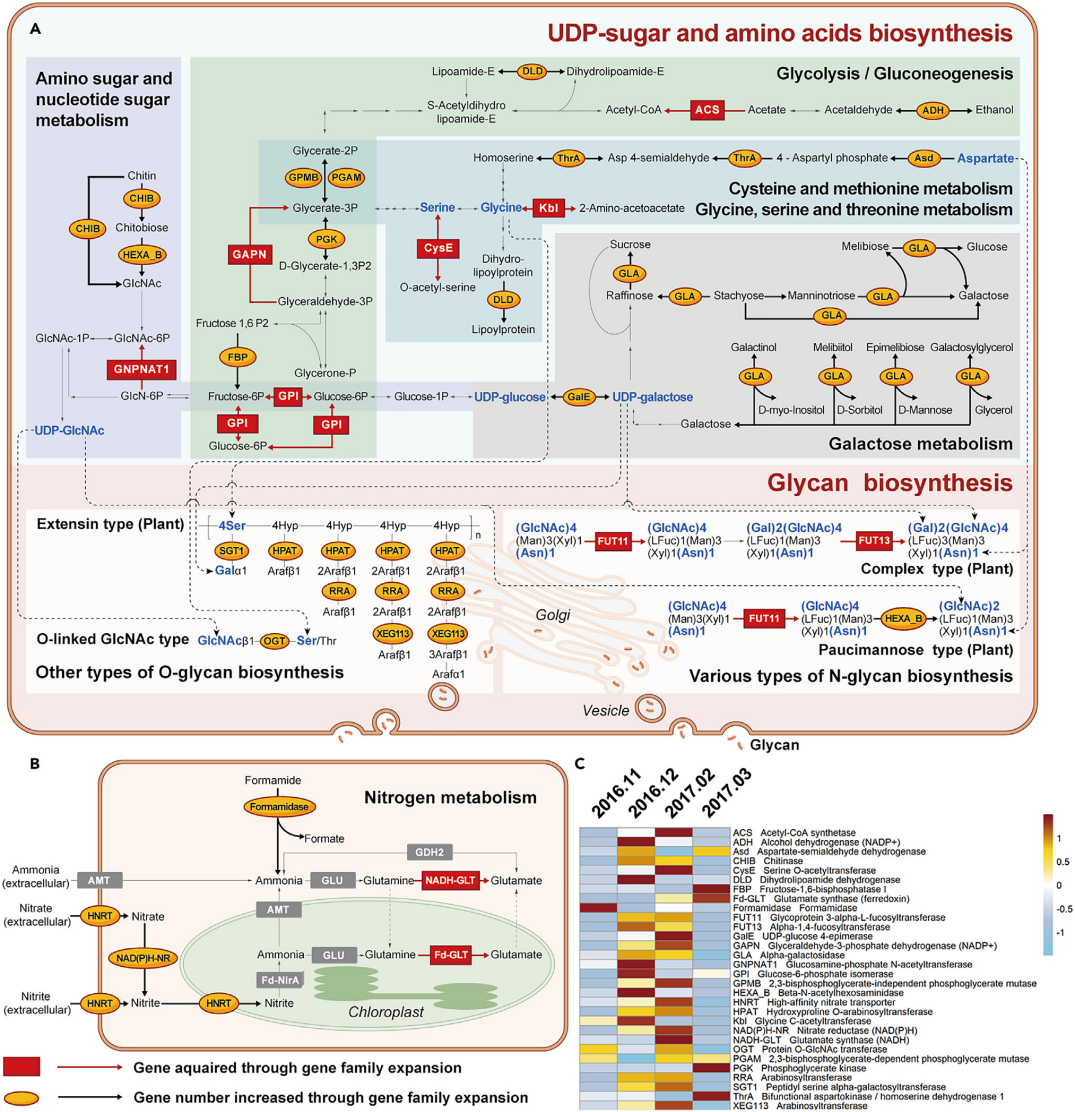
Illustration of *P. globosa* colony formation-associated biological pathways impacted by gene family expansion (A) Glycan precursors and glycan biosynthesis. (B) Nitrogen metabolism. (C) Differential gene expression during *P. globosa* bloom development. Araf, arabinofuranose; Asn, glutamine; Fuc, fructose; Gal, galactose; GlcN, glucosamine; GlcNAc, N-Acetyl-glucosamine; Hyp, hydroxyproline; Man, mannose; Ser, serine; Xyl, xylose.

Comparative analysis of *P. globosa* genomes and genome of related species revealed the expansion of 926 gene families, impacting 184 KEGG pathways, many of which are associated with colonial formation during *P. globosa* blooms, including various types of N-glycan biosynthesis (ko00513), and other types of O-glycan biosynthesis (ko00514). Gene family expansion also affected the biosynthesis of polysaccharide chains pathway (Figure 3A). For example, UDP-sugar biosynthesis pathway, amino sugar and nucleotide sugar metabolism (ko00520), glycolysis/gluconeogenesis (ko00010), galactose metabolism (ko00052), and the amino acid metabolism related pathways including cysteine and methionine metabolism (ko00270) and glycine (Table S3; Figure S5), serine and threonine metabolism (ko00260). Analysis of gene expression during bloom development revealed that the expanded genes showed enhanced expression when colonial densities were high (December, 2016-February, 2017), suggesting that the expansion of gene families might have facilitated gene expression, which in turn promoted colony formation, leading to bloom development.

In addition, gene family expansion in *P. globosa* also affected the nitrogen metabolism (ko00910) pathway (*p* < 0.05) (Figures 3B and S6), consistent with previous reports that *P. globosa* prefers NO_3_^-^ to ammonia,^43^^,^^44^ which is different from many other picoplankton species, which prefers ammonia. Furthermore, there was an expansion of nitrate transporter in *P. globosa* with thirteen copies of nitrate reductase genes identified in *P. globosa*, which is more than that in *Emiliania huxleyi*, which has 12 copies of nitrate transporter genes. During the *P. globosa* bloom recorded in the Beibu Gulf in 2016–2017, nitrate concentration recorded in December, 2016 was 3.96-fold higher than that recorded in February, 2016 (Figure 3C; Tables S4–S6). However, the expression of nitrate transporter was higher in February, 2017 (Figure 3C), suggesting that *P. globosa* has a strong preference for nitrate, consistent with previous reports.^43^^,^^44^

### Extensive *P. globosa* genomic variations may facilitate diverse adaptation

**Figure 4:**
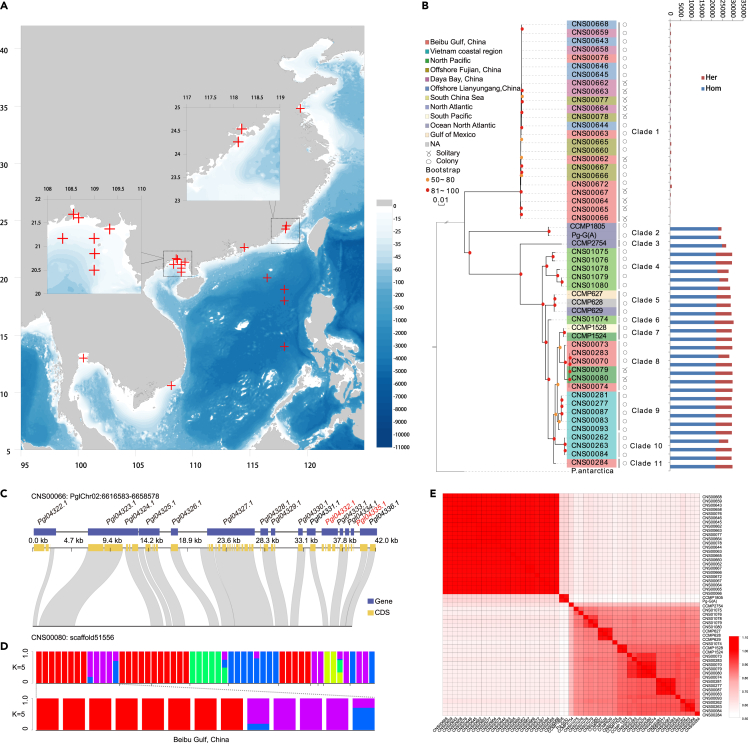
Genetic diversity and population genetics of *P. globosa* (A) Geographical locations of sampling sites and morphology for *P. globosa* strains. (B) Phylogenetic tree of 39 strains inferred from whole-genome SNPs, basing whole-genome resequencing results. (C) Pairwise comparison of a scaffold of the CNS00080 with its corresponding genomic region of reference strain CNS00066. Yellow rectangles represent coding sequences (CDSs), while blue rectangles represent PCGs. Gray ribbons represent corresponding genomic regions between these two strains. (D) Population structure of 13 strains isolated from the Beibu Gulf, with reference to *P. globsa* population in other regions. Population structure of *P. globosa* population, and the Beibu Gulf strain is shown separately. (E) Pairwise genomic sequence similarity of *P. globosa* strains.

To explore genomic variations in *P. globosa*, we isolated *P. globosa* strains from coastal regions in China (including the Beibu Gulf, Guangxi Province; Lianyungang, Jiangsu Province; Zhangzhou, Fujian Province; Daya Bay, Guangdong Province), Thailand, and Vietnam (Figure 4A). Phylogenetic analysis based on single nucleotide variations (SNVs) indicated that these *P. globosa* strains (including the Pg-A strain isolated from the North Sea of Europe) could be divided into 11 clades (Figure 4B), confirming high genetic diversity of *P. globosa*. Interestingly, one clade consisted of *P. globosa* strains isolated from many different geographical regions including the Beibu Gulf (Guangxi, China), the Daya Bay (Guangdong, China), Zhangzhou (Fujian, China), and the Lianyun Harbor (Jiangsu, China), suggesting that this clade is a “cosmopolitan”. In contrast, many other clades consisted of *P. globosa* strains collected from one or a small number of regions, suggesting stronger preference to unique ocean regions. For example, the second clade consisted of two *P. globosa* strains isolated from the North Sea, Europe, Clade 4 consisted of five *P. globosa* strains isolated from the South China Sea, and Clade 9 consisted of eight strains collected from the coast of Vietnam.

To appreciate the level of genomic variations among *P. globosa* strains, we carried out pairwise alignment analysis. Alignment of Illumina sequencing reads of a representative *P. globosa* strain in Clade 8 (CNS00080, which was isolated from the coast of Thailand) against the reference genome (CNS00066) revealed that only ∼60% of the reference genome was covered with a minimal 4-fold depths, suggesting major genomic differences between *P. globosa* strains. Altogether, 74,668 deletion events (≥ 50 bp in sizes) and 80,752 insertion events were identified. At least 382 protein coding genes were affected because they were entirely nested within the deletion events, many of which were annotated to contain zinc finger domains, suggesting potential functional differences between these strains. Figure 4C displayed a representative pairwise comparison of orthologous segments of the two genomes, illustrating substantial presence and absence variations (PAVs) in the *P. globosa* genome.

*P*. *globosa* strains isolated from the Beibu Gulf were found in three separate clades (clades 1, 8 and 11), suggesting the co-existence of *P. globosa* strains with high genetic diversity in the Beibu Gulf. Population analysis also indicated extensive admixture of *P. globosa* strains (Figure 4D). Pairwise genomic sequence similarity comparison of these *P. globosa* strains supported the existence of substantially different clades shown in the phylogenetic analysis (Figure 4E).

### Polymorphic rhodopsin genes in *P. globosa*

**Figure 5:**
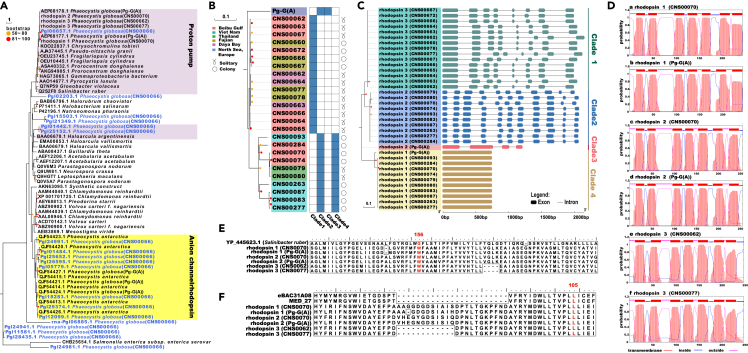
Rich and diverse proton pump-type rhodopsin genes in the *P. globosa* (A) Putative proton pump-type rhodopsin genes annotated in the reference *P. globosa* strain (CNS00066). (B) Putative proton pump-type rhodopsin genes annotated in different *P. globosa* strains. (C) Gene structures of proton pump-type rhodopsin genes annotated in *P. globosa* strains. (D) Transmembrane domains annotated in the proton pump-type rhodopsin genes in *P. globosa* strains. (E) Peptide sequence alignment of the putative ketocarotenoid-binding region of rhodopsin genes. Amino acids corresponding to Gly156 of *Salinibacter ruber* xanthorhodopsin are highlighted in red. (F) Peptide sequence alignment of rhodopsin genes with eBAC31A08. Amino acids corresponding to 105 of eBAC31A08 are highlighted in red.

Searching the *P. globosa* proteome identified 19 rhodopsin-like genes that may serve the function of photoreception.^45^ Comparative analysis of these putative rhodopsin-like genes suggested that three genes (*Pgl06657.1*, *Pgl01442.1*, and *Pgl25152.1*) encode putative proton pumps,^46^ while eight genes (*Pgl24991.1*, *Pgl01484.1*, *Pgl25652.1*, *Pgl26595.1*, *Pgl05779.1*, *Pgl18283.1*, *Pgl29374.1*, and *Pgl12099.1*) encode putative anion channel rhodopsin proteins, comparing to three ACRs reported in *P. globosa* recently^47^ (Figure 5A). *P*. *globosa* may be able to migrate over a longer range of marine space with these rhodopsin genes, whose long-wavelength light (∼590 nm) better penetrates biological tissue.^47^ Functions of other putative rhodopsin-like genes are currently unknown.

Among the three putative proton pumps, gene *Pgl06657.1* showed high similarity to two previously reported rhodopsin-like genes *AEP68177.1* (rhodopsin 1) and *AEP68178.1* (rhodopsin 2), putative proton pump genes identified in the Pg-G(A) strain of *P. globosa* (Figure 5A).^48^ Extensive search of a second proton pump gene in the reference genome (CNS00066) was not successful, suggesting that different *P. globosa* strains have different numbers of this type of proton pump gene. To test this hypothesis, we search for homologs of rhodopsin 1 and rhodopsin 2 in the genomes of *P. globosa* strains assembled using Illumina sequencing reads. As expected, the numbers of proton pump genes found in *P. globosa* strains were variable (Figure 5B). Based on their similarity and the numbers of introns contained in these genes, and their relative positions in the phylogenetic tree, these putative rhodopsin genes were classified into four clades (Figure 5C). Clade 1 rhodopsin genes, each of which contained eight exons and seven introns, were only found in the 15 *P. globosa* strains that contained single rhodopsin genes, including the reference strain CNS00066 (Figure 5C). Clade 2 rhodopsin genes, each of which contained 11 exons and 10 introns, were found to co-exist in 10 *P. globosa* strains that also contained Clade 4 intronless rhodopsin genes (Figure 5C). Clade 3 rhodopsin gene, which contained four exons and three introns, was found only in the Pg-G(A) strain, which also contained a Clade 4 intronless rhodopsin gene (Figure 5C). Prediction of *trans*-membrane domains revealed that each of the protein sequences encoded by these putative rhodopsin genes contained seven *trans*-membrane domains (7TM) as expected (Figure 5D).^45^ As a Trp (W) residue was found in position 156 in proteins encoded by these putative rhodopsin genes (Figure 5E), they can be classified as xanthorhodipsin Subgroup II (Vollmers et al., 2013). Furthermore, as a Leu (L) residue was found at the position 105 (Figure 5F), proteins encoded by these rhodopsin genes may be green light receptors (Man D, 2003).

## Discussion

Since the first description of genus *P. globosa* by G. Lagerheim in 1893,^13^ interests in species of this genus has been intensifying over time because these species play an important role in ocean carbon cycle,^2^ synthesize and metabolize DMSP that have profound impact on climate control,^5^ were found to cause harmful blooms with devastating consequences with both negative social and economic impacts,^49^ and most curiously, possess the remarkable heteromorphic life cycle that alternates between colonies of non-motile coccoid cells and solitary cells.^18^^,^^21^ As such, extensive research has been done on these cosmopolitan *Phaeocystis* species especially on *P. globosa*.^1^^,^^11^^,^^12^^,^^20^^,^^22^

Taking advantage of a *P. globosa* strain (CNS00066) with low bacterial contamination, and the newly established PacBio HiFi DNA sequencing platform coupled with Hi-C analysis methods, we were able to construct the first chromosome-scale genome assembly for *P. globosa*. The success of this project was not trivial, building upon many rounds of unsuccessful attempts, including construction of multiple genomic DNA libraries, and application of different sequencing and assembly strategies, partially due to the high bacteria content of colonial samples. The estimated genome size of *P. globosa* was 144.48 Mb, which was within the estimated *P. globosa* genome size range (109–200 Mb) reported previously.^31^ This *P. globosa* genome assembly (129.7 Mb) represents the first genome assembly in the phylum Haptophyta with chromosomal continuity (Table 1), and one of only 15 algal genomes among 85 reported algal genomes that achieved chromosome-scale, including six Chlorophyta species, five Ochrophyta species, three Cryptophyta species, and one Rhodophyta species (Table S1, data accessed at September, 14, 2021).

The ultimate success of this *P. globosa* genome project enabled us to appreciate for the first time the full-length chromosomes of *P. globosa*, some of which contained telomeric motif CCCTAA, highlighting the completeness of these chromosomes. Notably, *P*. *globosa* is only the second algal species whose complete complement of ribosomal gene clusters have been successfully resolved, after the red alga *Cyanidioschyzon merolae*, whose genome hosts only three copes of ribosomal gene clusters in two chromosomes.^50^ Furthermore, comparative analysis revealed two rounds of segmental genome duplication events in the evolutionary history of this species. These two rounds of segmental genome duplications may have fueled the evolution of *P. globosa* in reshaping its DMSP metabolism, cosmopolitan distribution, and solitary-colonial alternation of heteromorphic life history, which are analyzed below. The *P. globosa* represents only the second algal species after the oleaginous diatom *Fistulifera solaris* whose genome may have experienced extensive duplications.^51^ The identification of these two pathways (DYSB-dependent pathway and MT2-dependent pathway) fits well with the “Algae, bacteria and corals” pathway.^52^

The successful reconstruction of the *P. globosa* genome assembly also enabled us to look for answers to many of its remarkable role in ecology and its fascinating life cycle. First, comparative analysis of the *P. globosa* genome revealed the co-existence of dual DMSP biosynthesis pathways, which may suggest that *P. globosa* can utilize these pathways in response to different environmental conditions, which may explain why *P. globosa* has been known for its role in DMSP biosynthesis and degradation.^4^^,^^5^^,^^6^^,^^7^ While some algal species including dinoflagellates and prymnesiophytes utilized HiDP DMSP biosynthesis pathway with high production rates, others including diatoms utilized LoDP DSMP biosynthesis pathway with minimal production rates.^8^ Co-existence of HiDP and LoDP pathways have also been found in *P. antarctica*, and in a few other species *Chrysochromulina tobin*, *Sagittula stellate*, *Amorphus coralli*, and *Labrenzia aggregate*.^9^ However, *P. globosa* not only possesses the dual DMSP biosynthesis pathways, the gene responsible for DMSP degradation, *Alma*, showed copy number variations that it had different numbers of copies in different strains, which may make different strains different in degrading DMSP.

The polymorphism is not limited to the *Alma* genes. *P. globosa* encodes a large set of rhodopsin genes that may play essential role in photoreception, which may explain why radiation plays an important role in *P. globosa* bloom development.^11^ A recent study reported three channelrhodopsin homolog genes in *P. globosa* that encoded anion channels (ACRs) (Govorunova et al., 2020). The reference *P. globosa* genome (CNS00066) encoded 19 putative rhodopsin genes, including eight putative ACRs. Annotation of homologs of rhodopsin 1 and rhodopsin 2 in different *P. globosa* strains suggested the numbers of rhodopsin gene copies were highly variable among different strains. While some strains contained single homolog of this type of rhodopsin genes, many others encoded two homologs, all of which encoded proteins with 7TMs. These putative rhodopsin genes may enable *P. globosa* strains to adapt to various radiation levels. Although proton pump rhodopsin of haptophyte (*P. globosa*), diatoms and dinoflagellate clustered in the phylogenetic tree (Figure 5A), *P. globosa* proton pump rhodopsin were neither close to those of diatoms (which is believed to serve in response to iron limitation^46^), nor to those of dinoflagellates (which has been shown to respond to phosphate limitation^53^), suggesting that genes of these proton pump rhodopsin are evolutionarily distant. Thus, the ecological role of *P. globosa* proton pump rhodopsin remains to be ascertained.

Polymorphism was not limited to *Alma* genes and rhodopsin genes. Indeed, comparative analysis of 53 strains collected from various ocean regions revealed remarkably high genetic diversity. For example, only ∼60% of the reference genome (CNS00066) could be aligned by reads of the CNS00080 (which was isolated from coastal region of Thailand). This level of genomic differences was comparative to the intra-species difference observed in the coccolithophore *Emiliania huxleyi*.^39^ Interestingly, *P. antarctica* also showed high genetic diversity, as demonstrated by differential responses to iron and light conditions.^54^

The successful reconstruction of the *P. globosa* genome represents another major advance toward understanding its heteromorphic life cycle, its role in DMSP metabolism, and *Phaeocystis* bloom development. Analyses presented in this paper only highlighted a tiny portion of the value of the newly constructed *P. globosa* genome, which serves as a platform for many more extensive researches on *P. globosa* biology and ecology.

### Limitations of the study

In this study, we constructed chromosome-level genome assembly only for one *P. globosa* strain (CNS00066). As comparative analysis revealed substantial genomic differences between different *P. globosa* strains, chromosome-level genome assemblies of multiple representative *P. globosa* strains would be desirable. Such genome assemblies would enable better identification of strain-specific genomic differences, and better construction of *P. globosa* pangenome.

## STAR★Methods

### Key resources table

**Table:** 

REAGENT or RESOURCE	SOURCE	IDENTIFIER
**Phaeocystis globosa strains**		

*Phaeocystis globosa* strain	This study	CNS00066
*Phaeocystis globosa* strain	This study	CNS00668
*Phaeocystis globosa* strain	This study	CNS00659
*Phaeocystis globosa* strain	This study	CNS00643
*Phaeocystis globosa* strain	This study	CNS00658
*Phaeocystis globosa* strain	This study	CNS00076
*Phaeocystis globosa* strain	This study	CNS00646
*Phaeocystis globosa* strain	This study	CNS00645
*Phaeocystis globosa* strain	This study	CNS00662
*Phaeocystis globosa* strain	This study	CNS00663
*Phaeocystis globosa* strain	This study	CNS00077
*Phaeocystis globosa* strain	This study	CNS00664
*Phaeocystis globosa* strain	This study	CNS00078
*Phaeocystis globosa* strain	This study	CNS00644
*Phaeocystis globosa* strain	This study	CNS00063
*Phaeocystis globosa* strain	This study	CNS00665
*Phaeocystis globosa* strain	This study	CNS00660
*Phaeocystis globosa* strain	This study	CNS00062
*Phaeocystis globosa* strain	This study	CNS00667
*Phaeocystis globosa* strain	This study	CNS00666
*Phaeocystis globosa* strain	This study	CNS00672
*Phaeocystis globosa* strain	This study	CNS00067
*Phaeocystis globosa* strain	This study	CNS00064
*Phaeocystis globosa* strain	This study	CNS00065
*Phaeocystis globosa* strain	This study	CNS01075
*Phaeocystis globosa* strain	This study	CNS01076
*Phaeocystis globosa* strain	This study	CNS01078
*Phaeocystis globosa* strain	This study	CNS01079
*Phaeocystis globosa* strain	This study	CNS01080
*Phaeocystis globosa* strain	This study	CNS01074
*Phaeocystis globosa* strain	This study	CNS00073
*Phaeocystis globosa* strain	This study	CNS00283
*Phaeocystis globosa* strain	This study	CNS00070
*Phaeocystis globosa* strain	This study	CNS00079
*Phaeocystis globosa* strain	This study	CNS00080
*Phaeocystis globosa* strain	This study	CNS00074
*Phaeocystis globosa* strain	This study	CNS00281
*Phaeocystis globosa* strain	This study	CNS00277
*Phaeocystis globosa* strain	This study	CNS00087
*Phaeocystis globosa* strain	This study	CNS00083
*Phaeocystis globosa* strain	This study	CNS00093
*Phaeocystis globosa* strain	This study	CNS00262
*Phaeocystis globosa* strain	This study	CNS00263
*Phaeocystis globosa* strain	This study	CNS00084
*Phaeocystis globosa* strain	This study	CNS00284

**Deposited data**		

whole genome sequence data	This study	GWHBJCL00000000
Submission ID	This study	WGS025593
BioProject	This study	PRJCA009598
Biosample	This study	SAMC755508
Genome database		https://ngdc.cncb.ac.cn/gwh

### Resource availability

#### Lead contact

Further information and requests for resources and reagents should be directed to and will be fulfilled by the lead contact, Nansheng Chen (chenn@qdio.ac.cn).

#### Materials availability

*P. globosa* strains generated in this study are available upon request.

#### Data and code availability

• The whole genome sequence data reported in this paper have been deposited in the Genome Warehouse in National Genomics Data Center, Beijing Institute of Genomics, Chinese Academy of Sciences/China National Center for Bioinformation, under accession number GWHBJCL00000000 (submission ID: WGS025593; BioProject: PRJCA009598; Biosample: SAMC755508) that is publicly accessible at https://ngdc.cncb.ac.cn/gwh.
• The dataset is publicly accessible.
• Any additional information required to reanalyze the data reported in this paper is available from the lead contact upon request.
• This paper does not report original code.

### Method details

#### *P. globosa* strain isolation and culture; genome sequencing, assembly and annotation

The *P. globosa* strain CNS00066 was isolated from water samples collected in the Beibu Gulf, Guangxi Province during a *P. globosa* bloom in February, 2019. Individual cells were isolated from colonial forms and cultured individually. The strain CNS00066 does not form colonies in culture bottles under laboratory conditions. This strain was purified by selecting single cells for culture individually. The strain CNS00066 was selected for the reference genome project because it contained the lowest amount of bacterial contamination (3.85%) among over a dozen strains examined. provided an excellent material for preparing high-quality DNA for the genome project. DNA samples were sequenced using PacBio HiFi technology.

PacBio HiFi sequencing reads were assembled using FALCON (v0.2.2), followed by error correction and polishing using pilon (v1.22).^55^ The resulting contigs were assembled into chromosomes through Hi-C analysis using Juicer,^56^ which were further corrected using JucieBox. To evaluated quality of the assembled genome sequences, PacBio HiFi reads were aligned against the assembled genome sequences using minimap2 (with default parameters),^57^ followed by calculating reads alignment rates, percentage of coverage, and alignment depth distribution. To evaluate the accuracy of the assembled genome sequences, Illumina sequencing reads were aligned against the assembled genome sequences using BWA,^58^ followed by SNP calling and filtration using GATK.^59^ Homozygous and heterozygous SNPs were counted. BUSCO^60^ analysis was done to evaluate the completeness of the assembled genome.

Repeat content was annotated using two methods. The first method was a homology-based repeat discovery method using RepeatMasker and and RepeatProteinMask^61^ and the RepBase library (http://www.girinst.org/repbase). The second method was a *de novo* method using RepeatModeler^62^ and LTR-FINDER.^63^ We also predicted tandem repeats using TRF.^64^

Protein-coding genes (PCGs) were annotated using three methods. The first method was a homology-based method using PCGs of closely related organisms (*Emiliania huxleyi*, *Chrysochromulina tobin*, *Tisochrysis lutea* and *Chlamydomonas reinhardtii*) as references. The second was a *de novo* gene prediction method using three programs including Augustus, Genscan, and GlimmerHMM. The third method was transcriptome-based method using RNA-Seq data and Iso-Seq. Annotation results from these three methods were combined using MAKER.^65^ Finally, the gene set was functionally annotated using multiple protein databases (SwissProt, TrEMBL, KEGG, InterPro, GO, and NR). tRNA genes were annotated using tRNAscan-SE. rRNA genes were annotated using BLASTN. miRNA and snRNA genes were annotated using INFERNAL.^66^ The completeness of the annotated PCGs was evaluated using BUSCO with the database eukaryota_odb9.

#### Comparative genomics analyses

The *P. globosa* genome as compared against genome of a set of organisms including *Chrysochromulina tobin*, *Guillardia theta*, *Cyanidioschyzon merolae*, *Porphyridium purpureum*, *Chondrus crispus*, *Porphyra umbilicalis*, *Ectocarpus siliculosus*, *Chlamydomonas reinhardtii*, and the genome of *Naegleria gruberi* was used as the out group. Gene family clustering analysis was done using OrthoMCL.^67^ For better quality, comparison with identity <30% or coverage <50% was not included in subsequent analysis. For BLAST analysis, e-value threshold was set as 1e-5. For MCL clustering analysis, inflation parameter was set as 1.5.

Phylogenetic analysis and divergence estimation were done based on 145 single copy genes shared by the organisms included in this project. Multiple sequence alignment of the single copy genes was individually done using MUSCLE.^68^ These individual alignments were then concatenated (in phylip format), which was used to construct phylogenetic trees using Maximum Likelihood method available at RAxML.^69^ Divergence estimation was done by referring to the speciation times available at TimeTree (http://www.timetree.org/)^70^ and from literature, including *Chrysochromulina tobinii* vs. *P. globosa* (843-520 Mya), *P. globosa* vs. *Ectocarpus siliculosus* (1605-900 Mya), *Chondrus crispus* vs. *Porphyra umbilicalis* (1251-604 Mya), *Chondrus crispus* vs. *Porphyridium purpureum* (1251-604 Mya), *Chondrus crispus* vs. *Cyanidioschyzon merolae* (1487-806 Mya), *Guillardia theta* vs. *P. globosa* (1626-1189 Mya), *Guillardia theta* vs. *Naegleria gruberi* (2038-1094 Mya). Divergence estimation was done using r8s^71^ and mcmctree at PAML.^72^

#### Analysis of genome duplication

Three different methods were used to explore potential genome duplication events in the evolution of *P. globosa*. First, paralogs in *P. globosa* were searched using BLASTP (e-value < = 1e-5, coverage ≥ 50%), which were in turn used to search for pairs of gene blocks with high similarity. Second, pairs of gene blocks in *P. globosa* were searched for using MCScanX.^73^ Third, 4DTv and Ks values were calculated using KaKs_Calculator2.0^74^ and ParaAT,^75^ the distribution patterns of which were visualized using ggplot2. Relationships between pairs of gene blocks in *P. globosa* were displayed using Circos.^76^

#### Identification of genes involved in DMSP biosynthesis and degradation

Searching the *P. globosa* proteins using blastp (Evalue threshold = 1e-6) and the peptide encoded by *dsyb* (AOR83342) of *Labrenzia aggregata* as query) uncovered two candidate *DSYB* genes in *P. globosa* (*Pgl00289* and *Pgl15694*) with percentage identity of 37.6% and 28.2%, respectively. The O-methyltransferase domain (PF00891), which was the signature of proteins encoded by DSYB genes, was found in proteins encoded by both genes. We further searched for orthologs of DSYB genes in *Chrysochromulina tobin*, *Emiliania huxleyi*, *Aureococcus anophagefferens*, *Fragilariopsis cylindrus*, *Fistulifera solaris*, *Nannochloropsis gaditana*, *Phaeodactylum tricornutum*, *Thalassiosira oceanica*, and *Thalassiosira pseudonana*. We found two orthologs of DSYB in *Emiliania huxleyi* (XP_005772230 and XP_005781836), one in *Chrysochromulina tobin* (KOO32714), two in *Fragilariopsis cylindrus* (OEU17621and OEU16132), one in *Thalassiosira oceanica* (EJK51493). The O-methyltransferase domain (PF00891) was found in all of these genes except for XP_005781836 of *Emiliania huxleyi*. To search for genes encoding DSYB in *Phaeocystis antarctica*, we first assembled the *P. antarctica* genome using Illumina sequencing results, followed by searching the genome assembly using the two DSYB genes in *P. globosa* (Pgl00289 and Pgl15694) as queries. The results were further annotated using genewise, and two homologous genes were found, both of which contained the O-methyltransferase domain (PF00891).

Searching *P. globosa* proteins using TpMT2 gene of *Thalassiosira pseudonana* (XP_002291473) as query using BLASTP (Evalue threshold = 1e-6) identified a single homology PgMT2 (Pgl17043) with PID of 23.63%. A function domain Methyltransferase domain (PF08241) was found, followed by homologous gene finding using genewise.^77^

Searching *P. globosa* proteins using BLASTP (Evalue threshold = 1e-6) and seven Alma genes of *Emiliania huxleyi* (Alma1_XP_005784450.1, Alma2_XP_005763983.1, Alma3_XP_005793893.1, Alma4_XP_005778075.1, Alma5_XP_005776895.1, Alma6_XP_005786164.1, and Alma7_XP_005779316.1) as queries, identified three genes in *P. globosa*, which were *Alma1* (Pgl09637), *Alma2* (Pgl08660) and *Alma3* (Pgl17348), with PIDs 27.86%, 27.13%, and 25%, respectively. The function domain Asp_Glu_race superfamily (cl00518) was found in all *Alma* genes.

#### KEGG pathway analysis of samples collected during a *P. globosa* bloom

Surface water samples were collected at a sampling site that experienced *P. globosa* bloom (108°37′12″E, 21°10′12″N) at six time points including November, 2016, December, 2016, February, 2017, March, 2017, June, 2017, and August, 2017, which represented the initiation, development, and decay phases of the *P. globosa* bloom. RNA-Seq data were obtained by sequencing the RNA samples extracted from these water samples using Illumina X10 sequencing platform (Frasergen, Wuhan, China). On site observation identified *P. globosa* colonies in four water samples collected between November, 2016 and March, 2017. In contrast, no colonies were identified in the two samples collected in June, 2017 and August, 2017. The total sequence data were 51.83 Gb. Sequencing results from these six samples were mapped to the *P. globosa* genome, extracting transcripts corresponding to *P. globosa*.

#### Genomic variation analysis of *P. globosa* strains

The isolation and culture of *P*. *globosa* strains were described previously.^35^ A strain was called colonial if any colonial forms were observed in the culture, while a strain was called solitary if no colonial forms were observed (Figure 1A; Table S1). For Illumina sequencing, total nucleic acids were extracted using the OMEGA HP Plant DNA Mini Kit (Omega Biotek, Inc., United States) and quantified using a NanoDrop One spectrophotometer (Labtech International Ltd., Uckfield, United Kingdom). DNA samples of five *P. globosa* strains were prepared for whole genome sequencing.

For reads filtration of Illumina sequencing data, raw reads in FASTQ format were first processed through a series of quality control (QC) procedures: (1) removing reads with 10% unidentified nucleotides (N); (2) removing reads with >50% bases having Phred quality <5; (3) removing reads with adapters; (4) removing putative PCR duplicates generated by PCR amplification in the library construction process (read 1 and read 2 of two paired-end reads that were completely identical); (5) retaining only paired-end reads; (6) removing bases which at both ends of Read having Phred quality <20. For SNV calling and filtration, quality of these reads was assessed using FastQC v0.11.4, and then they were uniquely aligned to the strain CNS00066 reference genome using the BWA-MEM tool.^58^ Picard tool v1.94 was employed to mark duplicate reads in the previously mentioned alignments of the 39 different *P. globosa* strains to the reference CNS00066 genome. SNP calling was executed using Genome Analysis Toolkit (GATK).^59^ A high-confidence coding SNP dataset were obtained: (1) MAF (Minor Allele Frequency): 0.01; (2) Proportion of SNP covered samples to total samples: 80%.

#### Rhodopsin gene annotation and comparative analysis

Nineteen rhodopsin-like genes were identified using keywords in the annotated gene set. Phylogenetic analysis using amino acid sequences of these putative rhodopsin in *P. globosa* and rhodopsin genes from other species were constructed using Maximum Likelihood (ML) methods with 1000 bootstrap replicates in MegaX.^78^ Evolutionary models were selected using Model Selection, and the model was (LG + G) model. Annotation of proton pump type rhodopsin genes in *P. globosa* strains was carried out using GeneWise.^77^ Phylogenetic tree using amino acid sequences of proton pump type rhodopsin genes of different *P. globosa* strains were constructed by using Maximum Likelihood (ML) methods with 1000 bootstrap replicates. Appropriate evolutionary models were selected using Model Selection, and the model was (WAG + G) model. The numbers of predicted TMHs of in different *P*. *globosa* strains were predicted using thmhmm.^79^

## Supplemental information

Document S1. Figures S1–S6 and Tables S2–S6

Table S1. List of algal genomes that have been constructed and published, related to Figure 1
