# Supplementary material for: Chromosome-scale genome assembly reveals insights into the evolution and ecology of the harmful algal bloom species Phaeocystis globosa Scherffel
Source: iScience. 2024 Jul 25;27(8):110575. doi: 10.1016/j.isci.2024.110575 (PMC11347835; doi:10.1016/j.isci.2024.110575)
Supplement: Document S1. Figures S1–S6 and Tables S2–S6 [file mmc1.pdf]

iScience, Volume *27*

Supplemental information

Chromosome-scale genome assembly reveals insights into the evolution and ecology of the harmful algal bloom species *Phaeocystis globosa* Scherffel

Nansheng Chen, Qing Xu, Jianan Zhu, Huiyin Song, Liyan He, Shuya Liu, Xiuxian Song, Yongquan Yuan, Yang Chen, Xihua Cao, and Zhiming Yu

Supplementary Data

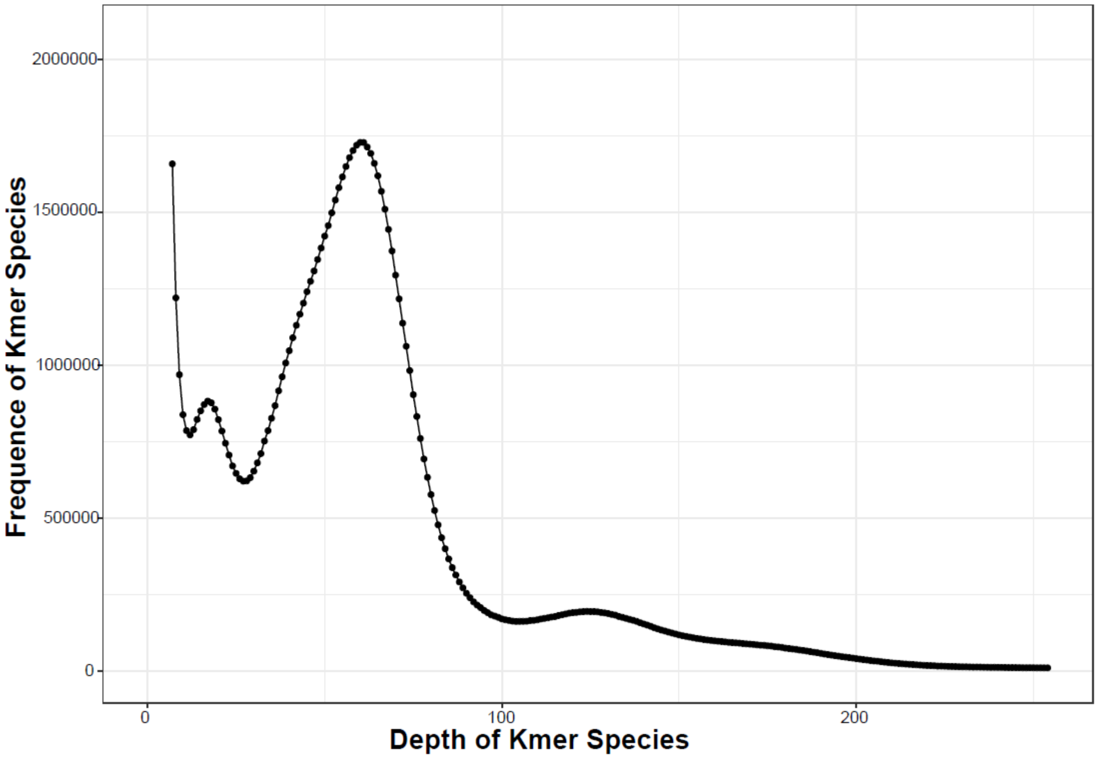

Fig. S1 Genome survey analysis of the *P. globosa* strain CNS00066, related to Figure 1A

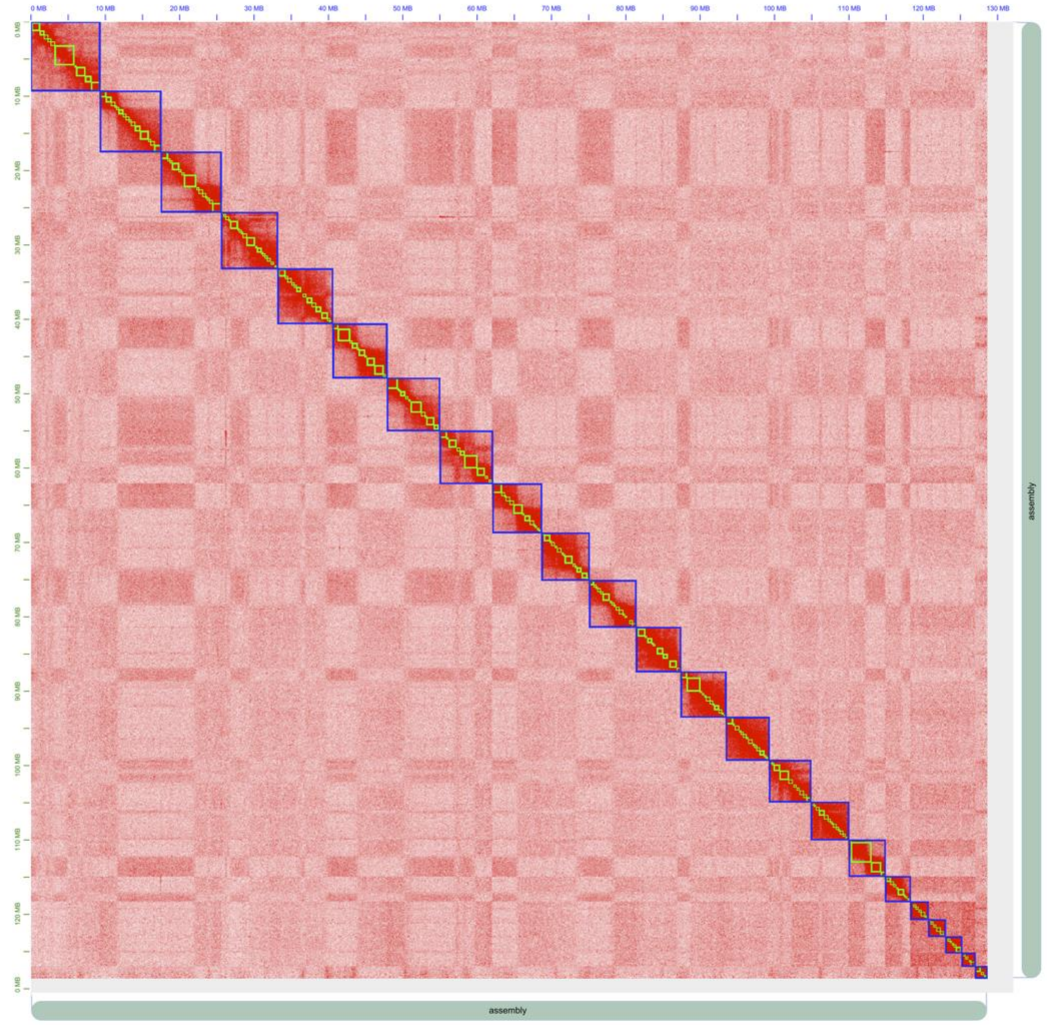

Fig. S2 Hi-C analysis of *P. globosa* genome assembly, related to Figure 1A.

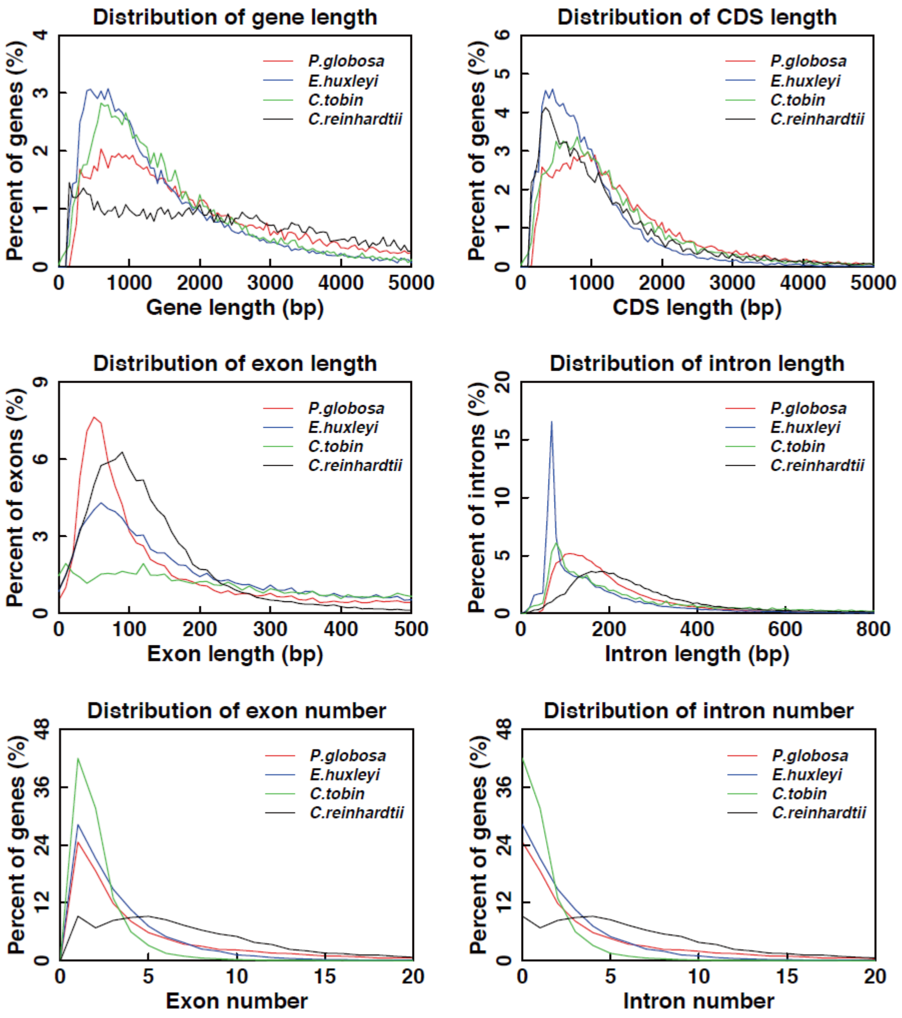

Fig. S3 Molecular features of protein-coding genes in *P. globosa*, related to Figure 1.

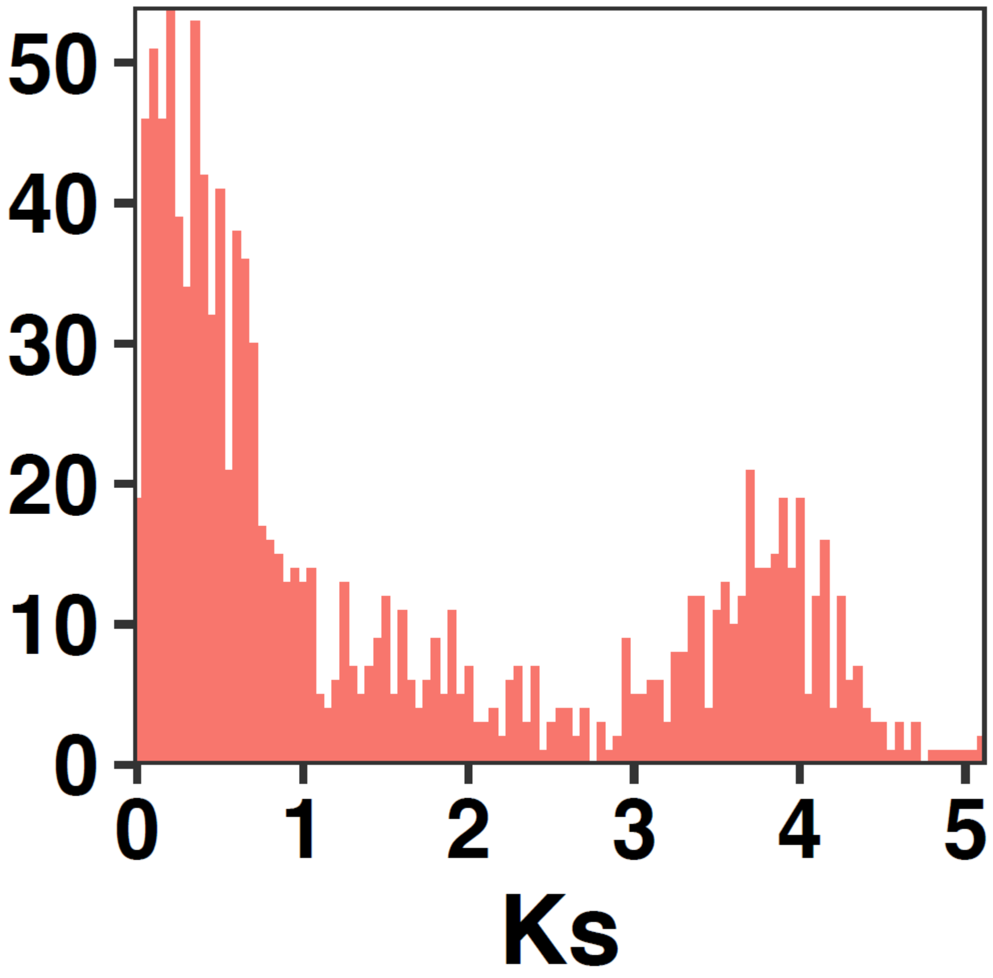

Fig. S4 Ks analysis of gene pairs in *P. globosa*, related to Figure 1.

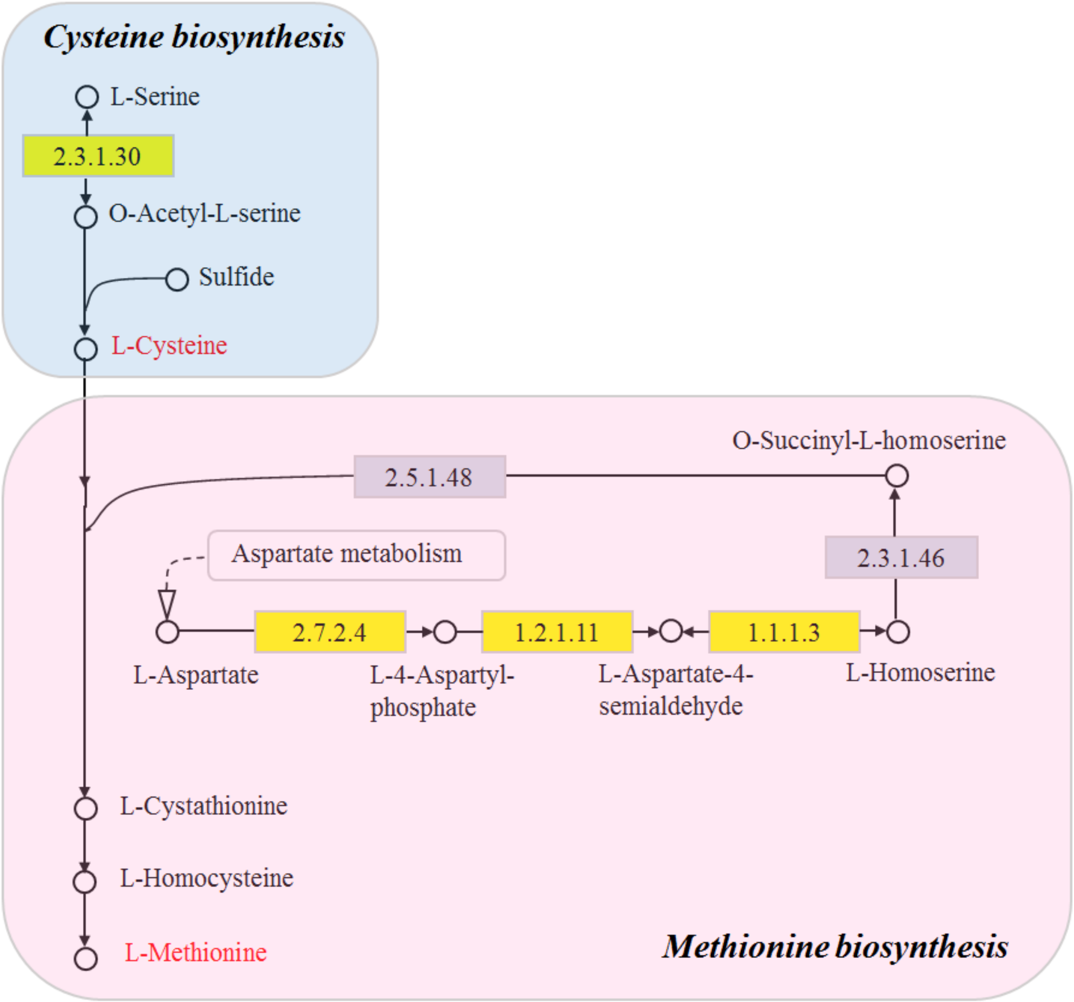

Fig. S5 Gene family expansion that affects the methionine biosynthesis pathway, related to Figure 3.

Yellow color indicates gene copy expansion.

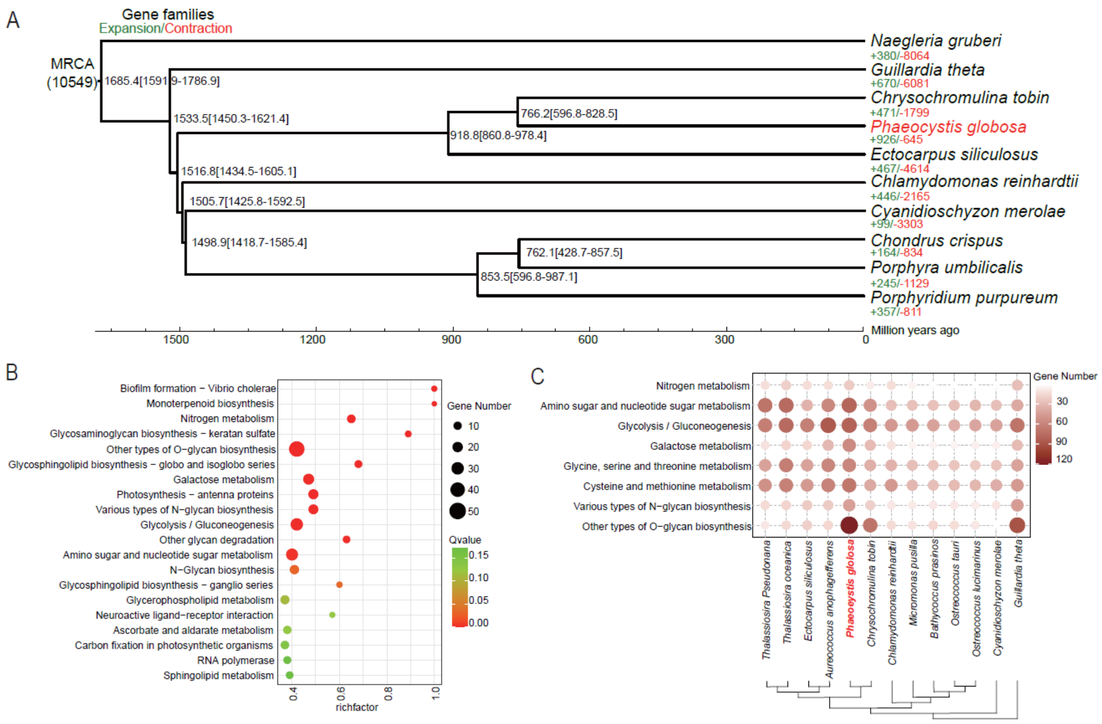

Fig. S6 Comparative genomics analysis of *P. globosa* and other eukaryotes, related to Figure 3. (A) Genome-wide analysis of expansions and contractions of gene families in *P. globosa* and other eukaryotes. Numbers on branches indicate the number of gene gains (+) or losses (−). (B) KEGG enrichment analysis of expansions genes (top20). The size of the circle represents the number of expansions genes. The color of the circle represents the Qvalue. (C) The number of genes involved in KEGG pathway of glucose metabolism and, nitrogen metabolism in *P. globosa* and its comparison with other algae.

Supplementary tables

Table S2 List of various types of TEs annotated in *P. globosa*, related to Figure 1.

**Type**	**Repbase TEs**		**TE protiens**		**De novo**		**Combined TEs**	
	Length (bp)	% in genome	Length (bp)	% in genome	Length (bp)	% in genome	Length (bp)	% in genome
DNA	5182491	4	5841	0	2437180	1.88	7551611	5.82
LINE	3324068	2.56	8161	0.01	4979763	3.84	8175067	6.3
SINE	333	0	0	0	0	0	333	0
LTR	798741	0.62	1105124	0.85	10487180	8.09	11389278	8.78
Other	170	0	0	0	0	0	170	0
Unknown	28243	0.02	0	0	3995148	3.08	4023374	3.1
Total	9334046	3.2	1113285	0.86	19462091	15.01	23588222	18.18

Table S3 Copy numbers of genes associated with methionine biosynthesis copy expansion in *P. globosa* and related organisms, related to Figure 3.

Ko gene	Gene name	Gene des	*Thalassiosira pseudonana*	*Thalassiosira oceanica*	*Ectocarpus siliculosus*	*Aureococcus anophagefferens*	*Emiliania huxleyi*	***Phaeocystis globosa***	*Chrysochromulina tobin*	*Chlamydomonas reinhardtii*	*Micromonas pusilla*	*Bathycoccus prasinos*	*Ostreococcus tauri*	*Ostreococcus lucimarinus*	*Cyanidioschyzon merolae*	*Guillardia theta*	
K00640	cysE	serine O-acetyltransferase [EC:2.3.1.30]	3	1	5	6	6	**5**	1	3	3	3	3	3	2	1	**rna-Pgl31076.1**<br>**rna-Pgl11205.1**<br>**rna-Pgl11206.1**<br>**rna-Pgl11855.1**<br>**rna-Pgl15973.1**
K00133	asd	aspartate-semialdehyde dehydrogenase [EC:1.2.1.11]	1	2	1	2	1	**3**	0	1	2	1	1	1	1	2	rna-Pgl32057.1<br>**rna-Pgl31902.1**<br>**rna-Pgl20552.1**
K12524	thrA	bifunctional aspartokinase/homoserine dehydrogenase [EC:2.7.2.4 1.1.1.3]	4	4	3	1	6	**5**	2	2	2	1	1	1	1	3	rna-Pgl31758.1<br>rna-Pgl31901.1<br>rna-Pgl22169.1<br>**rna-Pgl06780.1**<br>**rna-Pgl29344.1**

Note: The last column shows the numbers of copies of various genes in *P. globosa*.

Table S4. Six sets of metatranscriptomic data obtained in the Beibu Gulf, related to Figure 3.

Sample	Clean Reads Pairs	Length	Q20(%)	Q30(%)	GC(%)
2016.11	28,104,412	150;150	98.1;98.1	94.7;94.3	55.5;55.5
2016.12	30,927,772	150;150	98.2;97.5	94.7;92.7	57.3;57.3
2017.02	29,401,559	150;150	98.3;98.2	94.9;94.5	56.9;56.9
2017.03	30,515,633	150;150	98.1;97.7	94.7;93.4	57.7;57.7
2017.06	25,915,596	150;150	98.3;98.1	95.1;94.1	53.0;53.0
2017.08	27,887,097	150;150	97.5;97.0	93.5;92.3	56.8;56.8

Table S5. ***P. globosa*** **transcriptomic information extracted from six sets of metatranscriptome data, related to Figure 3.**

Total mapped reads represent numbers of clean reads that mapped to the reference genome. Uniquely mapped reads represent numbers of clean reads that mapped to the reference genome only at one site.

Sample	Total mapped reads	Uniq mapped reads	Total Gene	Expressed Gene
2016.11	110,647(0.39%)	63,688(0.23%)	32618	9504 (29.14%)
2016.12	1,325,198(4.28%)	1,096,857(3.55%)	32618	21970 (67.36)
2017.02	2,495,603(8.49%)	2,241,814(7.62%)	32618	25395 (77.86)
2017.03	292,644(0.96%)	130,417(0.43%)	32618	3275 (10.04)
2017.06	46,407(0.18%)	17,379(0.07%)	32618	2826 (8.66)
2017.08	84,938(0.30%)	29,513(0.11%)	32618	936 (2.87)

Table S6 The number and average size of colonies for the six samples, related to Figure 3.

	**2016.11**	**2016.12**	**2017.02**	**2017.03**	**2017.06**	**2017.08**
The number of colonies (/m^3)	——	113	10806	51	0	0
The average size of colonies（mm）	2.24	3.55	3.14	4.67	——	——
